# Influence of Polyvinylpyrrolidone Concentration on Properties and Anti-Bacterial Activity of Green Synthesized Silver Nanoparticles

**DOI:** 10.3390/mi13050777

**Published:** 2022-05-15

**Authors:** Raghad Zein, Ibrahim Alghoraibi, Chadi Soukkarieh, Mohammad Taher Ismail, Abdalrahim Alahmad

**Affiliations:** 1 Department of Physics, Faculty of Sciences, Damascus University, Damascus P.O. Box 30621, Syria; 2 Department of Basic and Supporting Sciences, Faculty of Pharmacy, Arab International University, Daraa P.O. Box 30621, Syria; 3 Department of Animal Biology, Faculty of Sciences, Damascus University, Damascus P.O. Box 30621, Syria; soukkarieh@gmail.com; 4 Department of Biochemistry and Microbiology, Faculty of Pharmacy, Arab International University, Daraa P.O. Box 30621, Syria; mt-ismail@aiu.edu.sy; 5 Institute of Technical Chemistry, Leibniz University Hannover, Callinstrasse 5, 30167 Hannover, Germany

**Keywords:** silver nanoparticles (AgNPs), *Eucalyptus*, polyvinylpyrrolidone, zeta potential, antibacterial

## Abstract

Environmentally green synthesis of stable polyvinyl pyrrolidone (PVP)-capped silver nanoparticles (PVP-AgNPs) was successfully carried out. The present study focused on investigating the influence of adding PVP during the synthesis process on the size, optical properties and antibacterial effect of silver nanoparticles produced. An aqueous extract of *Eucalyptus camaldulensis* leaves was used as a reducing agent. The effects of different PVP concentrations and reducing time on the synthesis of nanoparticles (NPs) were characterized by UV–Vis spectrophotometry, scanning electron microscopy (SEM), energy dispersive spectrum (EDX), Fourier transform infrared spectroscopy (FTIR), dynamic light scattering (DLS) and nano tracker analysis (NTA). The addition of PVP was studied. The prepared PVP-AgNPs were spherical with an average size of 13 nm. FTIR analysis confirmed that PVP protects AgNPs by a coordination bond between silver nanoparticles and both N and O of PVP. DLS results indicated the good dispersion of silver nanoparticles. PVP-AgNPs were found to be stable for nearly 5 months. Antibacterial studies through the agar well diffusion method confirmed that silver nanoparticles synthesized using PVP had no inhibitor activity toward Gram-positive and Gram-negative bacteria as opposed to silver nanoparticles prepared without adding PVP, which showed a significant antibacterial activity towards some of the tested pathogens.

## 1. Introduction

Noble metal nanoparticles, mainly silver nanoparticles (AgNPs) have been an attractive research subject due to their exceptional properties [1], such as biocompatibility, high surface area, quantum confinement, variable size, shape and structure (nanosphericals, nanorods, nanocubes and nanotriangles). AgNPs are extensively incorporated in various fields [2] such as in cosmetics [3], food packaging [4], bioengineering, biomedicine [5,6,7,8,9,10,11] (biosensing, anticancer, antimicrobial, antileishmanial, antioxidant, medical treatment, drug delivery and cell imaging), catalysis [12], antimicrobial paints [13], surface cleaners, air and water disinfection [14] and wastewater treatment [15]. Various methods were successfully reported for the synthesis of silver nanoparticles including chemical and physical routes [16]. However, most of the materials used in these methods are hazardous substances and harmful to both humans and the environment. Today, scientists and researchers explore the possibility of using natural materials as reducing agents for the synthesis of nanoparticles such as plant extracts [17] and live organisms [18] like bacteria, fungi [19] and yeast [20]. Adding a capping agent (i.e., Glycerol, sodium oleate, Polyethylene glycol (PEG). Polyvinyl alcohol (PVA) and Polyvinylpyrrolidone (PVP)) [21] during the preparation of silver nanoparticles controls the rate of growth, aid in preventing aggregation, enhance their properties and decrease their size [22]. PVP is one of the most popular polymeric materials used to encapsulate silver nanoparticles, sines it is non-toxic, biodegradable, biocompatible and temperature-resistant. PVP can serve as carriers of silver nanoparticles in silver-containing nanocomposites and drug-delivery systems. Ag-loaded nanocomplexes showed high antibacterial activity and low toxicity because of controlling the amount of released silver ions which varied according to the materials loaded on AgNPs [23,24]. Coated silver nanoparticles with PVP have great features and several potential applications such as purification of drinking water [25], food industries as packaging and color indicators [26,27], medical fields as health care and bioimaging [28] and pharmacology as drug delivery systems, controlled release of the drug and wound healing without any side effects [29]. Capping agents can stabilize a nanoparticle by different ways including electrostatic stabilization, steric stabilization, hydration forces, depletion and van der waals forces [30]. Polyvinylpyrrolidone has great affinity for silver due to its nitrogen and oxygen atoms. It acts as a capping agent through steric and electrostatic stabilization of amide groups of the pyrrolidone rings [30,31]. In this study, green synthesis of AgNPs was carried out using phytochemicals present in the aqueous extract of *Eucalyptus camaldulensis* leaves as reducing agent [32] and (PVP) has been used as a capping agent and stabilizer [33,34,35].

The goal of this study is investigating the influence of adding PVP during the green synthesis process on the size, optical properties and antibacterial effect of silver nanoparticles produced. This study highlights the absence of any antibacterial activity for silver nanoparticles prepared using PVP. To the best of our knowledge, there is no similar results have been reported earlier in the literatures [36,37]. This result support the benefit of coated silver nanoparticles with PVP to control the release ratio of silver ions and reduce the toxicity effect of AgNPs against living cells especially for medical and pharmacology applications as drug delivery systems and wound healing without any side effects [38]. The green synthesized AgNPs were characterized using various analyses such as UV–Vis spectrophotometry, scanning electron microscopy (SEM), energy dispersive spectrum (EDX), fourier transform infrared spectroscopy (FTIR), dynamic light scattering (DLS) and nano tracker analysis (NTA). The antibacterial activity of proposed AgNPs against different strains of pathogens (*Streptococcus* sp., *Enterococcus* sp., *Staphylococcus* sp. *Enterobacter* sp.) were assessed using agar well diffusion assays.

## 2. Materials and Methods

### 2.1. Materials

The materials, which were used in this work, are silver nitrate (AgNO_3_) as the metal precursor, polyvinylpyrrolidone (Mw = 10,000) as a capping agent and deionized water. All the chemicals were purchased from Sigma-Aldrich (Darmstadt, Germany).

### 2.2. Preparation of Plant Extract and Silver Nanoparticles

The method of preparation was mentioned in our previous work [39] with some modifications about adding PVP during the synthesis process. Briefly fresh and healthy *Eucalyptus camaldulensis* leaves were collected and cleaned with distilled water to remove all the dust and unwanted visible particles, then dried to constant weight at 60 °C for 2 h to remove the residual moisture. Dried leaves were powdered and stored until the extraction process. The aqueous extract was prepared by boiling 3 g of dried leaves with 30 mL of distilled water for 10 min. After that, the macerate was centrifuged at 9500 rpm for 5 min. Then the resulting supernatant was filtered by Whatman No.1 filter paper and stored in a dark container at refrigerator temperature. An aqueous solution (0.02 M) of silver nitrate was prepared by dissolving 0.068 g of AgNO_3_ in 20 mL of distilled water. Then 20 mL of different concentrations (5, 15, 50 mg/mL) of PVP solution has been added. The mixture was stirred for 15 min. After that, 4 mL of the prepared aqueous *Eucalyptus camaldulensis* leaves extract was added. The mixture was kept under stirring at room temperature for half an hour to ensure the homogeneous reduction process. The colorless mixture of silver nitrate and PVP changed to brownish-yellow within a short period (10 min), indicating the formation of AgNPs. After 24 h silver nanoparticles were separated by centrifugation at 9500 rpm for 10 min, and the residue was collected and washed four times with distilled water and once with ethanol. This operation was applied to get rid of any uncoordinated biological materials and the excess silver ions. The colloidal sample was prepared by adding distilled water to the washed silver nanoparticles and dissolving it using the ultrasonic bath for 15 min. 

### 2.3. Characterization Techniques

Several characterization techniques were used to study the properties of synthesized PVP-AgNPs. The optical properties of the synthesized silver nanoparticles were investigated by UV–Vis spectral study using a spectrophotometry Carry 5000. The aqueous extract was used as a baseline instead of water. The morphology of AgNPs was determined using high-resolution scanning electron microscopy (HRSEM) JEOL GmbH JSM-6700F SEM Tokyo, Japan) at an acceleration voltage of 20 KV with energy-dispersive X-ray spectroscopy analysis. The sample was prepared by dropping a small amount of colloidal nano-silver onto a carbon-coated graphite grid. Furthermore, the dried silver nanoparticles were analyzed by Fourier transform infrared spectroscopy (JASCO FT/IR-4200 Series, Tokyo, Japan) to identify the functional groups responsible for the synthesis of AgNPs. The particle size distribution and zeta potential of the prepared colloidal nano-silver were determined by means of a dynamic light scattering technique using Zetasizer Nano ZS from Malvern, Worcestershire, UK. For the zeta potential analysis, 1 mL of the sample was injected into the zeta cell, and the measurements were repeated three times after equilibration. Nano Tracker Analysis was used to record the Brownian motion of the particles in solution and to analyze the size distribution of nanoparticles. The sample was prepared by diluting 1 mM of AgNPs in ultrapure water (1:600) and injecting this into the NanoSight LM10 system (k, NanoSight Ltd., Amesbury, UK).

### 2.4. Antibacterial Assay

The antibacterial activity of the silver nanoparticles was assessed against different strains of pathogens (belonging to four genus: *Streptococcus* sp., *Enterococcus* sp., *Staphylococcus* sp. and *Enterobacter* sp. and one species: *Escherichia coli*) causing the acquired septicemia infection. These strains were isolated from the department of premature and newborns at a children’s hospital in Damascus and genotyped by Multiplex-PCR using specific primers. The bacteria were cultured on the nutrient agar medium and incubated at 37 °C for 24 h. One colony of the bacteria were selected using a sterile inoculating loop and suspended in 5 mL of physiological Serum. The turbidity of the bacterial suspensions was adjusted to the 0.5 McFarland standards. Sterile swabs were dipped into the inoculum tubes. Mueller Hinton agar plates were inoculated with bacteria by streaking the swabs. The antibacterial test was carried out via well diffusion method [40]. The wells (3 mm diameter) were made in each Petri plate with a cork borer and each well was filled with 100 µL of silver nanoparticles. Various concentrations (5, 12.5, 25 and 50 µg/mL) of silver nanoparticles prepared without using PVP have been used for antibacterial test and a concentration (12.5 µg/mL) of PVP-AgNPs has also been used. The zone of inhibition on the plates was recorded as an inhibition against the microbial species. The experimental results were expressed as mean ± standard deviation of three independent experiments. The statistical analysis of the data employed one way of variance (ANOVA) (significance level of *p* < 0.05), performed using Origin 8.5 software.

## 3. Results and Discussions

### 3.1. UV-Vis Spectroscopy Analysis

**Figure 1:**
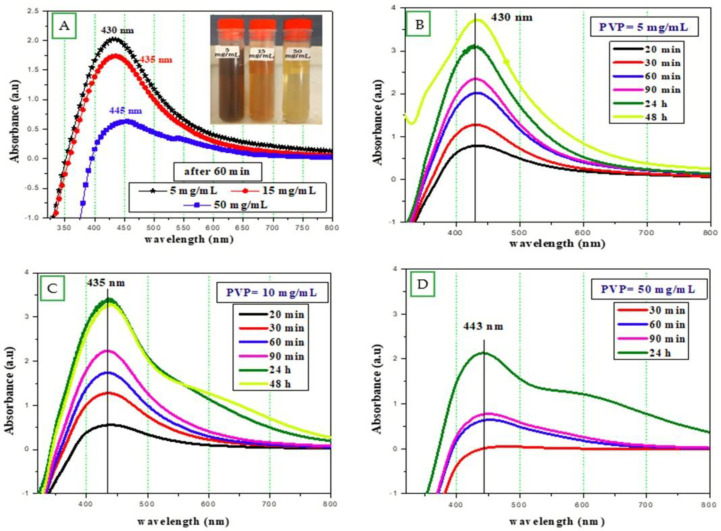
UV–Vis absorption spectra for PVP-AgNPs synthesized using an aqueous extract of *Eucalyptus camaldulensis* leaves (**A**) different concentrations of PVP (5, 15 and 50 mg/mL), (**B**–**D**) different reduction times at different concentrations of PVP 5, 15 and 50 mg/mL respectively.

After the addition of extract to the mixture of silver nitrate and PVP, the color of the solution changed from transparent to brownish-yellow. The color of the silver solution was found to depend on the concentration of PVP. The inset Figure 1A showed the colors changes with different concentrations of PVP after 10 min of reaction time. At low concentration (5 mg/mL), the color changed directly from light yellow to dark brown, while at high concentration the color changed to dark brown after half an hour. This slow change of color indicates the role of PVP as a capping agent. The formation of AgNPs was observed by the Localized Surface Plasmon Resonance (SPR) peak which can be influenced by the size, shape, aggregation, nature of the medium surrounding the nanoparticle and dielectric environment of the prepared nanoparticles [41,42]. Figure 1A illustrated absorption spectra for colloidal nanosilver synthesized at different concentrations of PVP (5, 15 and 50 mg/mL). The intensity of the SPR peak increased with decreasing the concentration. The sample at a low concentration of PVP has the highest yield. This demonstrated that a high concentration of PVP has limited the formation of AgNPs. According to Dagmara Malina et al. work [43], the high concentration of stabilizer leads to the formation of a dense polymeric network, which precludes the observation of nanostructures. Moreover, such a large amount of the stabilizer can prevent nanoparticles reactions with other compounds due to entanglement in the dense network of the stabilizer. This explains the low intensity of SPR peak at the high concentration of PVP. It is observed the blue shift of the maximum absorbance wavelengths (λ_max_) from 450 to 430 nm by decreases the concentration of PVP, which indicates the formation of nanoparticles of small size at a low concentration of PVP. The nitrogen and oxygen atoms in polar groups have a strong affinity for silver ions and that is the main reason for PVP protecting AgNPs [44]. It coordinates with silver ions and controls the size of nanoparticles by forming a capping layer surrounding them. PVP prevents the aggregation of Ag NPs via the repulsive forces that arise from the interaction of hydrophobic carbon chains with each other in the solvent [45].

The UV-Vis spectra for all samples synthesized using different reaction times and different concentrations of PVP are presented in Figure 1B–D. The gradual increase of SPR peak intensity with time indicates more AgNPs are formed. At a low concentration 5 mg/mL, the peak width (FWHM) values are (124, 125, 121, 122, 127 and 158 nm) at 20 min, 30 min, 60 min, 90 min, 24 h and 48 h respectively. These values are close to each other, which refers to the homogeneity of synthesized silver nanoparticles. There has been no change in the position of λ_max_ with time for all the different concentrations of PVP, which indicates the role of polyvinyl pyrroliydone as a stabilizer and capping agent which prevent aggregation of the nanoparticles over time. At high concentrations of PVP (15 and 50 mg/mL), a new shoulder peak appeared at approximately 600 nm after 24 h. This peak is thought to be due to the formation of low-symmetry nanoparticles in the colloid with increasing the reaction time. Low symmetry features cause nonuniform distribution of electron density, which affects the oscillation frequency and leads to a red-shift of resonance peak compared to spherical particles and the appearance of multiple peaks in the spectrum [46].

**Figure 2:**
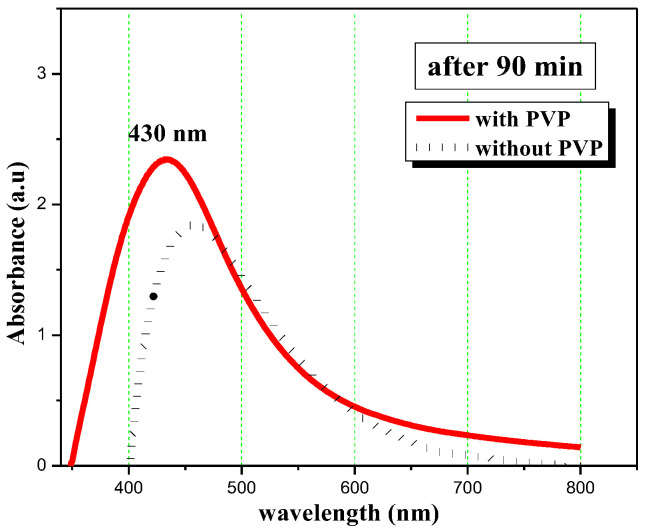
A comparison between the absorption spectra of nanosilver prepared by adding PVP (5 mg/mL) and silver nanoparticles prepared without adding PVP.

Figure 2 shows a comparison between the absorption spectra of nanosilver prepared by adding PVP (5 mg/mL) and silver nanoparticles prepared according to the method mentioned in our previous work [47] without adding PVP, after 90 min of reaction time. Adding PVP during the preparation process of silver nanoparticles affects the position of SPR peak and thus the size of synthesized nanoparticles and affects the yield of prepared silver nanoparticles which is clear from the intensity of the surface plasmon resonance peak.

### 3.2. Scanning Electron Microscopy and EDX Analysis

**Figure 3:**
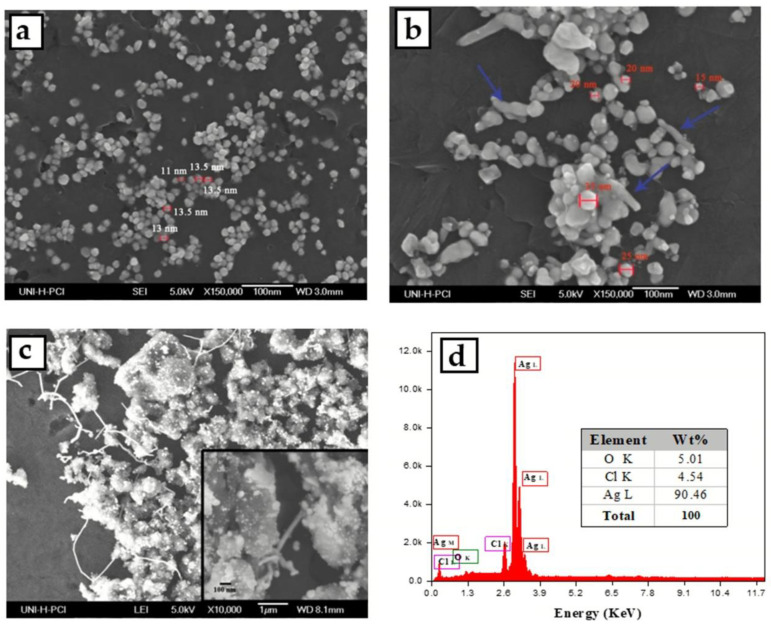
HRSEM images for PVP-AgNPs synthesized using an aqueous extract of *Eucalyptus camaldulensis* leaves at different concentrations of PVP (**a**) 5 mg/mL, (**b**) 15 mg/mL, (**c**) 50 mg/mL (**d**) elemental composition of the AgNPs synthesized using EDX detector.

SEM study was performed to investigate the size and shape of the prepared AgNPs. Figure 3 illustrates the SEM images of the silver nanoparticles prepared using different PVP concentrations (5, 15, 50 mg/mL). The NPs prepared at a low concentration of PVP were spherical, un agglomerated and uniformly distributed with a diameter of about 13 nm. which is also consistent with the narrow SPR peak at UV–VIS absorption spectrum. SEM images in Figure 3b are corresponding to the AgNPs prepared at a 15 mg/mL concentration of PVP which is revealed to the aggregation of spherical nanoparticles with a diameter ranging from 15 to 35 nm and forming some one-dimensional rod-like features, which might be due to the probable reason for the appearance of the small shoulder at 600 nm in the absorption spectrum. A high concentration of PVP in the solution leads to aggregation of silver nanoparticles, because of the destabilization of osmotic pressure (depletion flocculation phenomenon) [48] (see Figure 3c). These results are consistent with the redshift of the SPR peak to 443 nm. The high density of rod-like features could explain the increase in the intensity of the shoulder peak which appeared at approximately 600 nm in the absorption spectrum. Additionally, the elemental composition of the AgNPs synthesized via *Eucalyptus camaldulensis* leaves extract was determined using EDX detector shown in Figure 3d. The intense sharp signal obtained at 3 KeV shows that silver was the main element. The other weak signals for chlorine and oxygen indicate the presence of phytoconstituents on the surface of AgNPs. 

To sum up and according to UV-Vis spectrum and SEM study, the suitable concentration of PVP was 5 mg/mL for synthesis of homogeneous and small size silver nanoparticles using 0.02 mol/L of AgNO_3_ solution and volume ratio of ECL extract/AgNO_3_ solution: 2:10. 

### 3.3. FTIR Study

**Figure 4:**
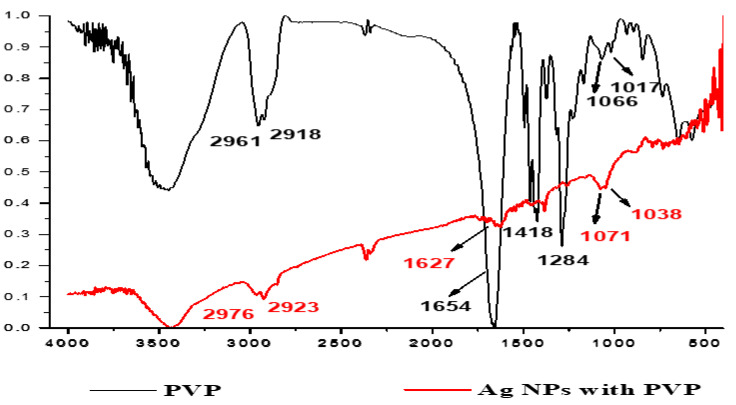
FTIR spectra of pure PVP and PVP-AgNPs.

Figure 4 represents FTIR spectra of pure PVP and PVP-AgNPs. In the spectra of pure PVP, the absorption peak locates at around 1654 cm^−1^ ascribed to the stretching vibration of C = O in the pyrrolidone group.

Compared with peaks of PVP-AgNPs that peak was shifted towards the lower wavenumber (1627 cm^−1^) due to the coordination between Ag and oxygen anion of the carbonyl group. In addition, absorption bands at 1418 cm^−1^ and 1284 cm^−1^ are due to the deformation vibration of CH_2_ in the skeleton chain of PVP and C–N bending vibration from the pyrrolidone structure. The slightly visible differentiating shifts in the spectra of pure PVP and silver colloids suggest coordination between silver nanoparticles and stabilizer, the peaks of C–N, at 1066 and 1017, were red-shifted to 1071 and 1038. This shift indicates that nitrogen electrons in pyrrolidine ring are involved in the formation of silver nanoparticles [43].

**Figure 5:**
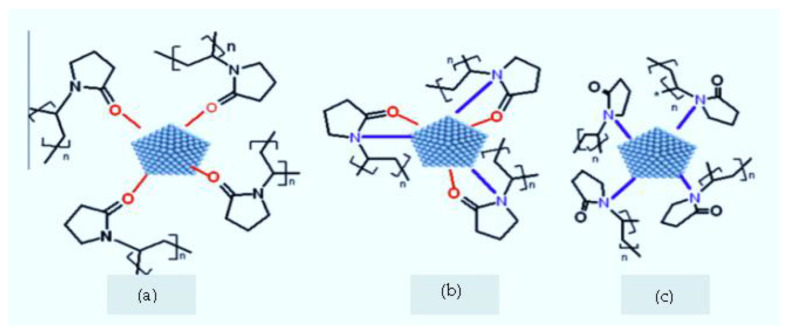
Three possible interactions of PVP molecule adsorbed onto a silver nanoparticle either by oxygen atoms only (**a**), both oxygen and nitrogen atoms (**b**) or by nitrogen atoms only (**c**).

The changes in the FTIR spectrum indicated that PVP protect silver nanoparticles by a coordination bond between nanoparticle and nitrogen and oxygen atoms of PVP. The three possible interactions of the PVP molecule adsorbed onto a silver nanoparticle are illustrated in Figure 5. The first one is interaction between the surface of silver nanoparticles and oxygen only, the second is related to the interaction with both nitrogen and oxygen of PVP, and the third is the possibility whereby nitrogen interacts with silver nanoparticles [49].

### 3.4. Dynamic Light Scattering Analysis

**Figure 6:**
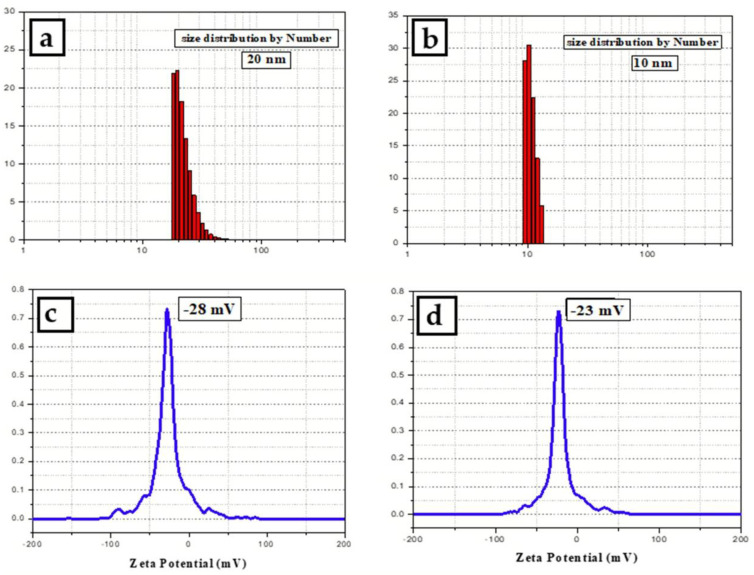
Particles’ size distribution histograms by number and zeta potential for silver nanoparticles synthesized using aqueous extract of *Eucalyptus camaldulensis* leaves (**a**,**c**) with adding 5 mg/mL of PVP and (**b**,**d**) without adding PVP.

The particle size distribution histogarms of silver nanoparticles synthesized with and without adding PVP were shown in Figure 6. The obtained size of PVP-AgNPs was around 20 nm. While the size for silver nanoparticles prepared without PVP was smaller than 10 nm. The presence of PVP as a capping agent surrounds the silver nanoparticles and forms a layer around them which affect the hydrodynamic diameter. By comparing the measured zeta potential values of the prepared silver nanoparticles as Figure 6c,d, we notice that the value for the samples prepared by adding PVP (−28 mV) is higher than those prepared without capping agent (−23 mV). This indicates that the addition of PVP makes the colloidal nanoparticles more stable. A negative value suggests the negatively charged surface of the nanoparticles, which causes an electrostatic repulsion force between the particles and thus increase the stability of the prepared colloidal.

### 3.5. NTA Analysis

**Figure 7:**
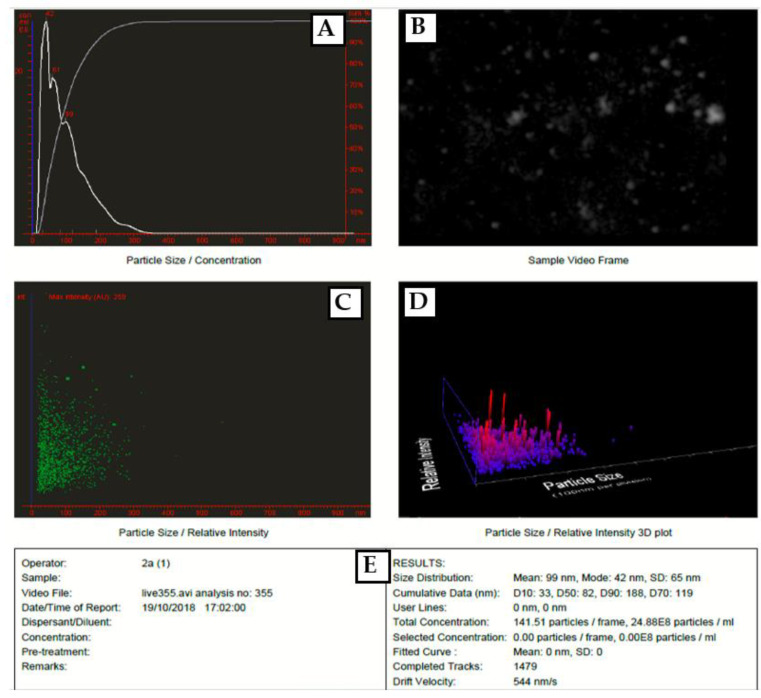
(**A**) particles size distribution, (**B**) A video frame, (**C**) 2D scattergram, (**D**) 3D plot and (**E**) results from NTA for PVP-AgNPs.

Nanoparticle Tracking Analysis measures particle size by video tracking. The brownian motion of each particle is followed simultaneously in real-time via video. An immediate idea of sample concentration and particle size is gained in seconds. Figure 7 showed a video frame and particle size distribution for AgNPs in solution. The mean size of silver nanoparticles synthesized *Eucalyptus camaldulensis* leaves extract was 99 nm and mode size 42 nm. PVP capping layer contributes to this increase in the particle size in the solution.

### 3.6. Stability of AgNPs

**Figure 8:**
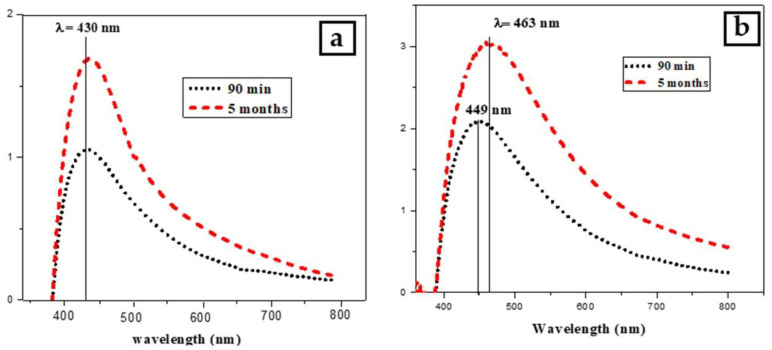
A comparison of Uv-Vis spectra for freshly and after 5 months silver nanoparticles, (**a**) prepared with PVP, (**b**) prepared without PVP.

The ability to store green-synthesized NPs for later use without changing their properties is beneficial to many applications. The stability of silver nanoparticles was investigated by monitoring the color of the reaction mixture and measuring the absorption spectra. Figure 8 indicates a comparison of absorption spectra for PVP-coated and uncoated AgNPs after 5 months. No evident change was observed in the position and width of absorbance peak for PVP-AgNPs which confirms the main role of PVP in increasing the stability by preventing aggregation of Ag nanoparticles. While the red shifted and broaden width SPR peak of uncoated AgNPs indicates the aggregation and sedimentation of nanoparticles.

### 3.7. The Antibacterial Activity of AgNPs

**Figure 9:**
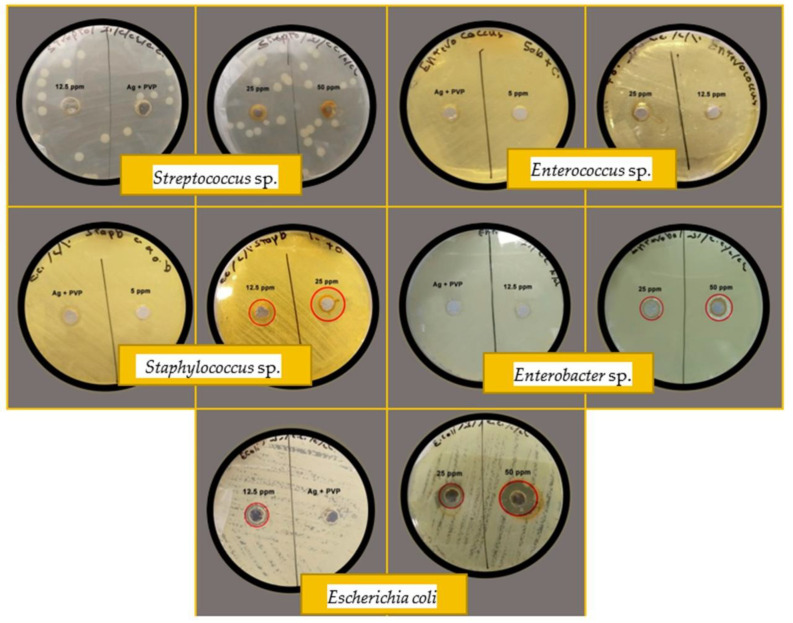
Antibacterial effect of PVP-AgNPs at concentration 12.5 µg/mL and silver nanoparticles synthesized without PVP at different concentrations: 12.5, 25, 50 µg/mL.

The antibacterial potential of silver nanoparticles has been tasted towards Gram-negative and Gram-positive pathogenic species via the agar well diffusion process (see Figure 9). The maximum diameter of the inhibition zone for different concentrations of silver nanoparticles is listed in Table 1. It has been shown that silver nanoparticles synthesized using an aqueous extract of *Eucalyptus camaldulensis* leaves and PVP as a stabilizing agent had no antibacterial properties because there was no zone of inhibition on the agar plates. This result conflicts with previous works that study the antibacterial properties of silver nanoparticles capped with PVP [36,50]. The absence of antibacterial activity is related to the formation of PVP layer around silver nanoparticles, which prevented the release of silver ions. Nevertheless, the antibacterial activity of PVP-AgNPs can be enhanced by changing the surrounding conditions to increase the solubility of the PVP and thus control the level of the released ions and enhance the antibacterial effect. On the other hand, the lack of the antibacterial activity of prepared PVP-AgNPs is consistent with other work reported earlier by Kaur et al. [23]. They improved the bactericidal efficacy by conjugation the silver nanoparticles with different classes of antibiotics. Where, the drug–AgNPs conjugates enhance the release of silver ions which attribute towards the synergetic effect.

AgNPs prepared using only *Eucalyptus camaldulensis* extract without adding PVP showed a significant inhibition towards some pathogens belonging to genus: *Staphylococcus* sp. and *Enterobacter* sp. and one species: *Escherichia coli*. While they have no bacterial activity against pathogens belonging to *Streptococcus* sp. or *Enterococcus* sp. even at a high concentration of 50 µg/mL.

AgNPs were observed to be more effective against Gram-negative bacteria (*Escherichia coli*) when compared to Gram-positive bacteria (*Staphylococcus* sp.), which might be due to the differences in bacterial pathogen’s membrane structures. The antibacterial effect is mainly due to the silver nanoparticles and not the organic materials of plant extract used during the synthesis process because the samples were washed five times after preparation. The bactericidal effect of AgNPs produced from *Eucalyptus camaldulensis* could be attributed to their small size and high surface to volume ratio, allowing them to interact very closely with bacterial membranes. The exact mechanism by which silver nanoparticles act as an antimicrobial has not been known. Based on current literatures [51,52], the proposed hypotheses on the action of silver nanoparticles as antibacterial have been summarized as follows: (i) silver nanoparticles can penetrate cell walls to cause structural changes to the membrane or increase its permeability. (ii) intracellular penetration of AgNPs; (iii) generation of reactive oxygen species (ROS) which damage the cell membrane and generate pores on the cell wall, resulting in cell death; (iv) Ag^+^ ions can interact with the thiol groups of many vital enzymes and protein, which can ultimately lead to cell death; (v) modulation of intracellular signal transduction pathways towards apoptosis. Critical parameters of AgNPs, such as shape, size, size distribution, surface charge, ion release, concentration and colloidal state, can all influence the antibacterial properties of AgNPs.

## 4. Conclusions

Very stable colloidal silver nanoparticles have been synthesized using an inexpensive, eco-friendly and green method. Adding polyvinylpyrrolidone as a stabilizing agent during the synthesis process reduces the size of nanoparticles and produces a more stable colloidal nanosilver in comparison to uncoated nanoparticles. PVP capping layer negatively affects the antibacterial properties of silver nanoparticles. There has no inhibition zone against any tested pathogens. Silver nanoparticles prepared using only *Eucalyptus camaldulensis* extract without adding PVP showed a significant inhibition towards some pathogens belonging to genus: *Staphylococcus* sp. and *Enterobacter* sp. and species *Escherichia coli*. This result opens new prospects for using polyvinyl pyrrolidone to control the silver ions release rate and thus control the dose to achieve the desired bactericidal effect and avoid toxicity. Finally, in the future, there is the requirement to understand the mechanism of Ag+ ions releas from PVP-AgNPs and compare its cytotoxicity effects with uncoated silver nanoparticles in mammalian cells.

## Figures and Tables

**Table 1:** Maximum diameters of inhibition zone for different concentrations of silver nanoparticles.

Bacterial Strains	Inhibition Zones in (mm)			
	AgNPs			AgNPs + PVP
	12.5 µg/mL	25 µg/mL	50 µg/mL	12.5 µg/mL
*Streptococcus* sp. Gr^+^	0.0 ± 0.0 ^c^	0.0 ± 0.0 ^c^	0.0 ± 0.0 ^c^	0.0 ± 0.0 ^c^
*Enterococcus* sp. Gr^+^	0.0 ± 0.0 ^c^	0.0 ± 0.0 ^c^	0.0 ± 0.0 ^c^	0.0 ± 0.0 ^c^
*Staphylococcus* sp. Gr^+^	16.2 ± 0.6 ^a^	19.8 ± 0.5 ^b^	N.A. *	0.0 ± 0.0 ^c^
*Enterobacter* sp. Gr^−^	0.0 ± 0.0 ^c^	14.4 ± 0.6 ^a^	16.5 ± 0.9 ^b^	0.0 ± 0.0 ^c^
*Escherichia coli* Gr^−^	14.3 ± 0.9 ^a^	15.4 ± 1.0 ^b^	23.3 ± 1.5 ^d^	0.0 ± 0.0 ^c^

Values are the mean ± standard deviation (S.D.) of three replicates. According to one way ANOVA test, S.D. within a column followed by different letters is significantly different at *p* ≤ 0.05 level. * (N.A.) means the concentration has not been applied.

## Data Availability

The study did not report any data.
